# Separation of Diamagnetic and Paramagnetic Susceptibility Sources in Liver Using DECOMPOSE‐QSM

**DOI:** 10.1002/nbm.70434

**Published:** 2026-11-06

**Authors:** Asli Alpman, Jingjia Chen, Jorge Elías Junior, Carlos Ernesto Garrido Salmon, Fuhua Yan, Hongjiang Wei, Chunlei Liu

**Affiliations:** ^1^ Department of Electrical Engineering and Computer Sciences University of California, Berkeley Berkeley California USA; ^2^ Bernard and Irene Schwartz Center for Biomedical Imaging, Department of Radiology New York University Grossman School of Medicine New York New York USA; ^3^ Center for Advanced Imaging Innovation and Research (CAI2R), Department of Radiology, New York University Grossman School of Medicine New York New York USA; ^4^ Department of Medical Imaging, Hematology and Clinical Oncology, FMRP University of São Paulo Ribeirão Preto Brazil; ^5^ InBrain, Department of Physics, FFCLRP University of São Paulo Ribeirão Preto Brazil; ^6^ Department of Radiology, Ruijin Hospital, School of Medicine Shanghai Jiao Tong University Shanghai China; ^7^ School of Biomedical Engineering Shanghai Jiao Tong University Shanghai China; ^8^ The National Engineering Research Center of Advanced Magnetic Resonance Technologies for Diagnosis and Therapy (NERC‐AMRT) Shanghai Jiao Tong University Shanghai China; ^9^ Helen Wills Neuroscience Institute University of California, Berkeley Berkeley California USA; ^10^ Department of Neuroscience University of California, Berkeley Berkeley California USA

**Keywords:** DECOMPOSE‐QSM, liver fibrosis, liver iron concentration (LIC), liver iron overload, quantitative susceptibility mapping (QSM)

## Abstract

Quantitative susceptibility mapping (QSM) enables noninvasive assessment of liver iron based on its paramagnetic properties. However, the paramagnetic contribution of iron can be confounded by the competing diamagnetic sources within the same voxel, particularly collagen in fibrotic tissue. Magnetic source separation techniques can disentangle these opposing susceptibility effects within a voxel, potentially enabling independent detection of iron and collagen. In this study, we conducted an initial evaluation of DECOMPOSE‐QSM using abdominal multi‐echo MRI data from a small patient cohort with normal to moderate liver iron overload (*N* = 19) and varying degrees of fibrosis (*N* = 3). Source‐separated paramagnetic susceptibility showed a stronger correlation with liver iron concentration (*R* ^2^ = 0.86, *p* < 0.05), whereas conventional QSM exhibited a weaker correlation (*R* ^2^ = 0.28, *p* = 0.08) in patients with normal to moderate iron overload. Furthermore, preliminary results in a small fibrotic cohort demonstrated that source‐separated diamagnetic susceptibility exhibit trends comparable to conventional QSM, suggesting that the method preserves sensitivity to fibrosis‐related tissue components.

## Graphical abstract

Separating subvoxel paramagnetic and diamagnetic susceptibility sources in the liver enables noninvasive assessment of iron overload and fibrosis. Using DECOMPOSE‐QSM, we disentangle iron‐related paramagnetic effects from collagen‐related diamagnetic contributions within the same voxel. Source‐separated paramagnetic susceptibility shows stronger correlation with liver iron concentration than conventional QSM, while the diamagnetic component provides sensitivity to fibrotic changes. These findings support susceptibility source separation as an approach for improved noninvasive liver tissue characterization.

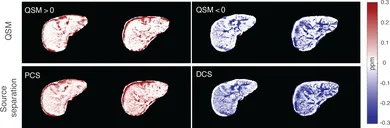

## 1 Introduction

Liver iron overload is the excess iron accumulation in the liver, which can result from hereditary iron overload, nonalcoholic/alcoholic fatty liver disease, and other conditions [1, 2]. Liver iron concentration (LIC) is essential for monitoring and managing iron overload and for assessing the risk of complications, including fibrosis and cirrhosis [3, 4, 5]. While biopsy is the gold standard for quantifying liver iron, its accuracy depends heavily on the sample taken, making it susceptible to sampling errors [6, 7]. Additionally, patient acceptance of liver biopsy is low due to high costs and risks such as pain and bleeding. Once the overload is diagnosed, regular monitoring of disease progression is required; however, repeated biopsies are impractical due to the associated risks and costs [8].

Magnetic resonance imaging (MRI) provides a non‐invasive alternative for liver iron quantification [9, 10, 11, 12, 13, 14, 15]. MRI‐based methods include R_2_ relaxometry [16, 17, 18, 19, 20], R_2_* relaxometry [21, 22, 23], and quantitative susceptibility mapping (QSM) [24, 25, 26, 27, 28, 29, 30, 31], all of which have been extensively validated and demonstrate strong correlations with LIC. However, these methods can be confounded by collagen accumulation in the liver due to fibrosis [32, 33, 34, 35], a wound‐healing response that often coexists with iron overload [36]. QSM has the potential to distinguish between them due to the opposing effects of diamagnetic collagen and paramagnetic iron on susceptibility measurements. However, since collagen and iron often coexist within the same imaging voxel, their phase effects can partially cancel each other out, complicating their separation using QSM. To address this challenge, previous studies have leveraged the magnetic anisotropy of collagen [37], a property absent in iron, or combined R_2_* measurements with QSM to improve fibrosis prediction accuracy [38].

Recently proposed susceptibility source separation methods based on QSM have the potential to separate paramagnetic and diamagnetic susceptibility [39, 40, 41, 42, 43, 44, 45]. These techniques employ a biophysical model of signal evolution to estimate paramagnetic and diamagnetic susceptibilities within a voxel by fitting measured signal to the model. By enabling the separation of paramagnetic and diamagnetic components within a voxel, these methods hold potential for application in the liver, where they could differentiate diamagnetic collagen from paramagnetic iron, aiding in the diagnosis of both iron overload and fibrosis. A recent ex vivo liver study applied susceptibility source separation and demonstrated its potential for discriminating histologic fibrosis stages [46].

In this work, we applied the DECOMPOSE‐QSM [39] method to identify paramagnetic and diamagnetic sources within a voxel to distinguish iron from collagen in vivo in the liver. While most source separation methods including DECOMPOSE‐QSM have been applied in the brain [47, 48], application in the body is further complicated by the fat chemical shift. We applied DECOMPOSE‐QSM to multi‐echo GRE abdominal MR images from three cohorts: healthy controls, an iron quantification cohort with liver iron concentrations ranging from normal to moderate overload, and a fibrotic cohort with varying stages of fibrosis. In the healthy and fibrotic cohorts, both fat‐saturated and non‐fat‐saturated acquisitions were obtained, enabling evaluation of the post‐processing fat‐water separation algorithm using fat‐saturated images as ground truth. The resulting paramagnetic and diamagnetic susceptibility maps provided enhanced contrast relative to conventional bulk QSM maps. Preliminary results from this work were presented at the 2024 ISMRM Annual Meeting [49].

## 2 Materials and Methods

### 2.1 Data Acquisition

Data were retrospectively obtained from three cohorts: a healthy control cohort, a cohort with iron quantification, and a cohort with liver fibrosis. All subjects either provided written informed consent under an approved study protocol, or consent was waived for the retrospective analysis of anonymized clinical data.

#### 2.1.1 Healthy Control Cohort (HC)

Fat‐saturated and non‐fat‐saturated breath‐hold abdominal images were acquired from 7 healthy control subjects using a 1.5‐T Siemens Aera scanner with a 3D FLASH multi‐echo GRE sequence using the following parameters: TE = [1.3, 3.9, 7, 10, 13.1, 16.1] ms, TR = 18 ms, bandwidth 1.08 kHz, flip angle = 10°, FOV = 299 × 399 mm, a spatial resolution of 2.08 × 2.08 × 5 mm, and 40 slices. The total acquisition time per subject was approximately 21 s without fat saturation and 24 s with fat saturation. CAIPIRINHA with an acceleration factor of 3 was used for both acquisitions. Fat suppression was achieved using the vendor‐provided quick fat saturation (Q‐fat sat) technique.

#### 2.1.2 Iron Quantification Cohort (IQC)

Abdominal breath‐hold MRI images from 12 subjects were acquired using a 1.5 T Siemens Aera scanner with a 3D FLASH multi‐echo GRE sequence, employing the following parameters: TE_1_/spacing/TE_6_ = 1.44/1.36/8.24 ms, TR = 10 ms, bandwidth 1.06 kHz, flip angle = 6°, FOV = 285 × 380 mm, spatial resolution of 1.7 × 1.7 × 5 mm and 44 slices. LIC values for all subjects were measured using R2‐based FerriScan. The total acquisition time per subject was approximately 15 s and 4/5 partial Fourier acquisition in the phase‐encoding direction was used to reduce acquisition time.

#### 2.1.3 Fibrotic Cohort (FC)

Fat‐saturated and non‐fat‐saturated breath‐hold abdominal images from three subjects with fibrosis were obtained on a 1.5 T Philips scanner using a 3D T1‐FFE multi‐echo GRE sequence with the following specifications: TE_1_/spacing/TE_12_ = 3.9/0.9/13.8 ms, TR = 18 ms, bandwidth 2.3 kHz, flip angle = 5°, FOV = 560 × 560 mm, spatial resolution of 2.5 × 2.5 × 5 mm, 40 slices and ProSet binomial (121) fat saturation with SENSE 2 × 3. Fibrosis staging for these subjects was determined based on clinical evaluation and MR images. The total acquisition time per subject is approximately 16 s with and without fat saturation.

### 2.2 DECOMPOSE‐QSM

The DECOMPOSE‐QSM method models signal evolution within a voxel using a three‐pool signal model consisting of a paramagnetic component, a diamagnetic component, and a neutral susceptibility medium [39]. The paramagnetic component is characterized by its volume fraction $\left(C_{+}\right)$, volume susceptibility $\left(\chi_{+}\right)$, and transverse relaxation rate $\left(R_{2,+}^{*}\right)$. Similarly, the diamagnetic component is defined by its volume fraction $\left(C_{-}\right)$, volume susceptibility $\left(\chi_{-}\right)$, and transverse relaxation rate $\left(R_{2,-}^{*}\right)$. The reference susceptibility medium is represented with its volume fraction $\left(C_{0}\right)$, a neutral volume susceptibility $\left(\chi_{0}=0\right)$, and a transverse relaxation rate $\left(R_{2,0}^{*}\right)$. The transverse relaxation rates are linked to their respective susceptibilities using the assumption of static dephasing, which leads to following signal model [39]:

(1) $$\begin{array}{c} S\left(t;C_{+},C_{-},C_{0},\chi_{+},\chi_{-},R_{2,0}^{*}\right)=C_{+}e^{-\left(a\chi_{+}+R_{2,0}^{*}+i\frac{2}{3}\chi_{+}\gamma B_{0}\right)t}\\ \;+C_{-}e^{-\left(-a\chi_{-}+R_{2,0}^{*}+i\frac{2}{3}\chi_{-}\gamma B_{0}\right)t}+C_{0}e^{-R_{2,0}^{*}t} \end{array}$$

where $B_{0}$ is the magnetic field strength, γ is the gyromagnetic ratio, and $a=\frac{2\pi }{9\sqrt{3}}\gamma B_{0}$. This signal model captures nonlinear phase evolution over time. Each component can be interpreted as a complex exponential with a distinct angular frequency and decay rate. The superposition of these components can produce a nonlinear phase trajectory over time.

The DECOMPOSE‐QSM algorithm estimates key parameters, including volume fractions, susceptibilities, and the reference relaxation rate through an alternating minimization method that separates the problem into linear and nonlinear optimization tasks. To accurately characterize the paramagnetic and diamagnetic components by accounting for both amplitude decay and phase evolution, metrics called paramagnetic component susceptibility (PCS) and diamagnetic component susceptibility (DCS) are calculated from the DECOMPOSE‐QSM outputs as follows [39]:

(2) $$\mathrm{PCS}=\frac{\sum_{t}∠\left(C_{+}e^{-\left(a\chi_{+}+R_{2,0}^{*}+i\frac{2}{3}\chi_{+}\gamma B_{0}\right)t}+\left(C_{0}+C_{-}\right)e^{-R_{2,0}^{*}t}\right)}{\frac{2}{3}\gamma B_{0}\sum t}$$

(3) $$\mathrm{DCS}=\frac{\sum_{t}∠\left(C_{-}e^{-\left(-a\chi_{-}+R_{2,0}^{*}+i\frac{2}{3}\chi_{-}\gamma B_{0}\right)t}+\left(C_{0}+C_{+}\right)e^{-R_{2,0}^{*}t}\right)}{\frac{2}{3}\gamma B_{0}\sum t}$$

Thresholded susceptibility maps with only positive and negative QSM values were constructed for comparison with PCS and DCS, respectively.

### 2.3 Fat Signal Removal Method

To eliminate fat chemical shift, non‐fat saturated images were processed using a fat‐water separation method based on a graph‐cut algorithm, incorporating a six‐peak fat and a single T_2_* decay model [50]. Subjects with fat‐water swaps within the liver were excluded from further analysis.

The fat‐water separation algorithm separates the input MR image $\left(s_{\text{nonfatsat}}\right)$ into fat‐only $\left(s_{\mathrm{fat}}\right)$ and water‐only $\left(s_{\mathrm{wat}}\right)$ components under the assumption of water‐only phase being linear with echo time due to local frequency shift. However, this assumption may not fully capture phase evolution in biological tissue, where compartments with different local frequency offsets and relaxation properties, as well as tissue microstructure and the spatial organization and orientation of susceptibility sources, can cause the signal phase to deviate from a linear dependence on echo time [39, 51, 52]. Therefore, we compared two approaches for deriving phase images for QSM reconstruction.

 
**Linear‐phase water images** ($s_{\mathrm{wat}}$): direct outputs of the fat‐water separation algorithm. Their phase evolves linearly over time and is attributed solely to local field inhomogeneity (B_0_).
 
**Nonlinear‐phase water images** ($s_{\text{nonfatsat}}-s_{\mathrm{fat}}$): created by subtracting the fat‐only image from the original input image. Unlike linear‐phase water images, these capture both local field inhomogeneity (B_0_) and additional phase components that evolve nonlinearly over time.

### 2.4 QSM Processing

After eliminating fat chemical shift, the resulting phases were unwrapped using a Laplacian‐based method [53]. Two tissue masks were generated: one is for the entire abdominal region for QSM reconstruction and another including only the liver for DECOMPOSE‐QSM. The liver mask was automatically segmented using nnU‐Net [54] and manually refined later. Lastly, the tissue phase was extracted with background phase removal using V‐SHARP [55].

To establish a robust zero‐susceptibility reference, both subcutaneous and visceral fat were utilized, as relying solely on subcutaneous fat was not feasible in subjects with a thin subcutaneous fat layer. Fat regions were segmented by selecting voxels with a fat fraction greater than 0.9 and R_2_* values below 300 s^−1^ to exclude regions affected by fat‐water swaps [56]. To ensure homogeneous susceptibility within the fat reference, the STAR‐QSM algorithm [57] was implemented with a regularization approach analogous to the CSF‐based regularization used in MEDI+0 [58]:

(3) $$\begin{array}{c} \chi =\underset{\chi }{\text{argmin}}\|\;\text{FT}⁻1\left(D\cdot \text{FT}\left(\chi \right)\right)-\varphi \;\|₂+\lambda ₁\|\chi \|₁\\ \;+\lambda ₂\|\;\chi \left(M_{\mathrm{fat}}\right)-\;\bar{\chi \left(M_{\mathrm{fat}}\right)\;}\|₂ \end{array}$$

where χ is the susceptibility, $D=1/3-k_{z}^{2}/k^{2}$ is the dipole kernel in Fourier domain, φ is the normalized frequency, $M_{\mathrm{fat}}$ is the fat mask, $\chi \left(M_{\mathrm{fat}}\right)$ is the susceptibility only within the fat region, and $\bar{\chi \left(M_{\mathrm{fat}}\right)\;}$ represents the mean susceptibility within the fat mask. $\lambda_{1}$ is the regularization constant for sparsity and $\lambda_{2}$ is the regularization constant for fat homogeneity. $\lambda_{1}$ was set to 1e‐6 for sparsity [57] and $\lambda_{2}$ was empirically set to 0.5. Calculated susceptibility maps for each echo, combined with the multi‐echo magnitude image, were input into the DECOMPOSE‐QSM algorithm.

### 2.5 Quantitative and Qualitative Analysis

#### 2.5.1 Region of Interest (ROI) Selection

To investigate correlations between PCS, DCS, and QSM values, liver ROIs were defined. Regions with the lowest local standard deviation, indicating spatial homogeneity, were selected as candidate ROIs. Homogeneous regions were identified by applying MATLAB *stdfilt* function to the R_2_* map, computing the local standard deviation within a 5 × 5 moving window. The final ROIs collectively covered 5% of the total liver volume and were manually reviewed to exclude visible vessels, bile ducts, and imaging artifacts.

#### 2.5.2 Statistical Tests and Analyses

Bland–Altman analysis is performed for linear‐phase and nonlinear‐phase water images to evaluate which fat signal removal strategy yields source separation results more consistent with the fat‐saturated reference. Robust linear regression with bisquare weighting (*fitlm* in MATLAB) was performed to assess correlations between LIC measured by FerriScan and QSM, PCS, and DCS metrics.

## 3 Results

### 3.1 Effect of Fat Signal Removal Method on DECOMPOSE‐QSM

Figure 1 includes a schematic illustrating the fat signal removal methods, as well as magnitude, QSM, PCS, and DCS maps for a representative subject (Subject 3 in FC dataset: FC3) with extensive fibrosis. While fat‐saturated (reference) and nonlinear‐phase water images show highly similar paramagnetic and diamagnetic distributions, linear‐phase water images exhibit diminished paramagnetic intensity and increased diamagnetic signal. These results suggest that nonlinear‐phase water images maintain higher fidelity to the fat‐saturated reference than linear‐phase water images.

**FIGURE 1:**
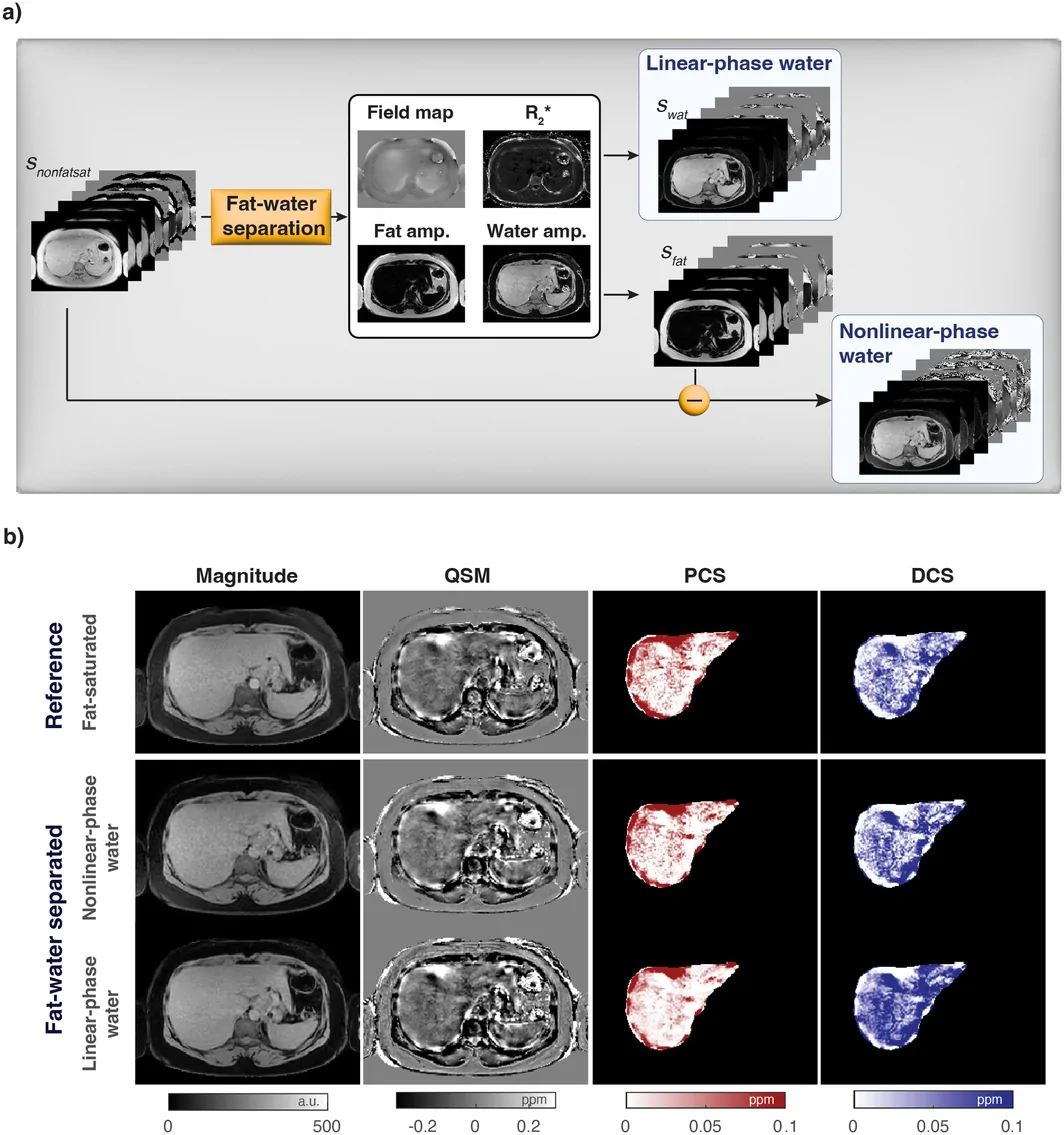
(a) Schematic illustrating the generation of linear‐phase and nonlinear‐phase water images. Linear‐phase water images are obtained directly from the fat‐water separation algorithm, whereas nonlinear‐phase water images are generated by echo‐wise complex subtraction of the estimated fat signal from the original non‐fat‐saturated signal. (b) Comparison of QSM, PCS, and DCS maps from a representative subject with extensive fibrosis (Subject FC3) across three fat signal removal methods: (1) fat‐saturated acquisition (reference; top row), (2) nonlinear‐phase water image (middle row), and (3) linear‐phase water image (bottom row).

Figure 2 presents Bland–Altman analyses of PCS and DCS, comparing fat signal removal techniques against fat‐saturated acquisitions as the reference standard in the HC and FC datasets, which included both fat‐saturated and non–fat‐saturated acquisitions. In the HC dataset, measurements derived from nonlinear‐phase water images showed strong agreement with the reference, with minimal mean bias for PCS (0.009 ± 0.009 ppm; limits of agreement (LoA): −0.009 to 0.027 ppm) and DCS (0.008 ± 0.008 ppm; LoA: −0.007 to 0.023 ppm). In contrast, linear‐phase water images showed a consistent negative bias, indicating systematic underestimation of both parameters, with mean biases of −0.021 ± 0.028 ppm (LoA: −0.028 to −0.014 ppm) for PCS and −0.079 ± 0.034 ppm (LoA: −0.145 to −0.013 ppm) for DCS.

**FIGURE 2:**
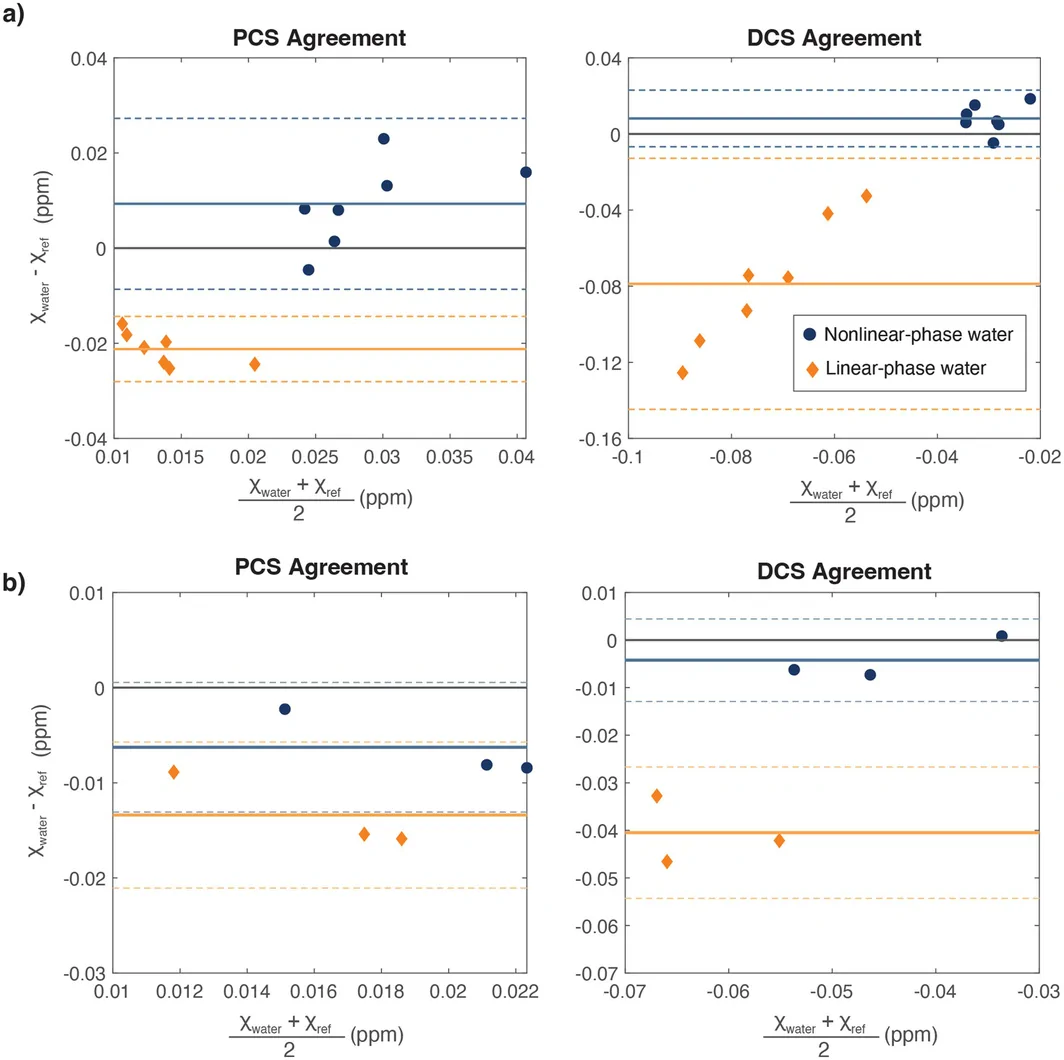
Bland–Altman analysis of PCS and DCS was performed to assess the agreement between susceptibility values obtained using the two fat signal removal approaches $\left(\chi_{\text{water}}\right)$ and those obtained from the fat‐saturated acquisition $\left(\chi_{\mathrm{ref}}\right)$, which was used as the reference. Results are shown for (a) the healthy cohort (HC) and (b) the fibrotic cohort (FC), with PCS presented in the left column and DCS in the right column. Nonlinear‐phase water results are shown as blue circles, whereas linear‐phase water results are shown as orange diamonds. For each method, the vertical axis represents the difference in susceptibility relative to the fat‐saturated reference, and the horizontal axis represents the mean susceptibility of the corresponding method and reference measurement. The solid line indicates the mean bias, while the dashed lines indicate the 95% limits of agreement.

Similar trends were observed in the FC dataset. Nonlinear‐phase water images again exhibited low bias, with mean biases of −0.006 ± 0.003 ppm (LoA: −0.013 to 0.001 ppm) for PCS and −0.004 ± 0.004 ppm (LoA: −0.013 to 0.004 ppm) for DCS. Linear‐phase water images continued to exhibit systematic negative bias, with mean biases of −0.013 ± 0.004 ppm (LoA: −0.021 to −0.006 ppm) for PCS and −0.040 ± 0.007 ppm (LoA: −0.054 to −0.027 ppm) for DCS. Across both datasets, linear‐phase water images consistently underestimated PCS and DCS and exhibited larger negative bias compared to nonlinear‐phase water images.

### 3.2 Liver iron Concentration Correlation With DECOMPOSE‐QSM

Figure 3 shows representative images from IQC dataset, including magnitude images, R_2_* maps obtained from the fat‐water separation algorithm, and QSM for four subjects. LIC measured by FerriScan for these subjects are 0.7, 1.9, 3.8, and 5.3 mg/g dry tissue, respectively. Increased $R_{2}^{*}$ values are evident in IQC3 and IQC4 subjects, consistent with their higher iron concentrations. Fat‐water swaps are noticeable, particularly in the subcutaneous fat regions. The QSM maps exhibit homogeneous susceptibility values across both subcutaneous and visceral fat, reflecting the effects of the applied regularization QSM inversion.

**FIGURE 3:**
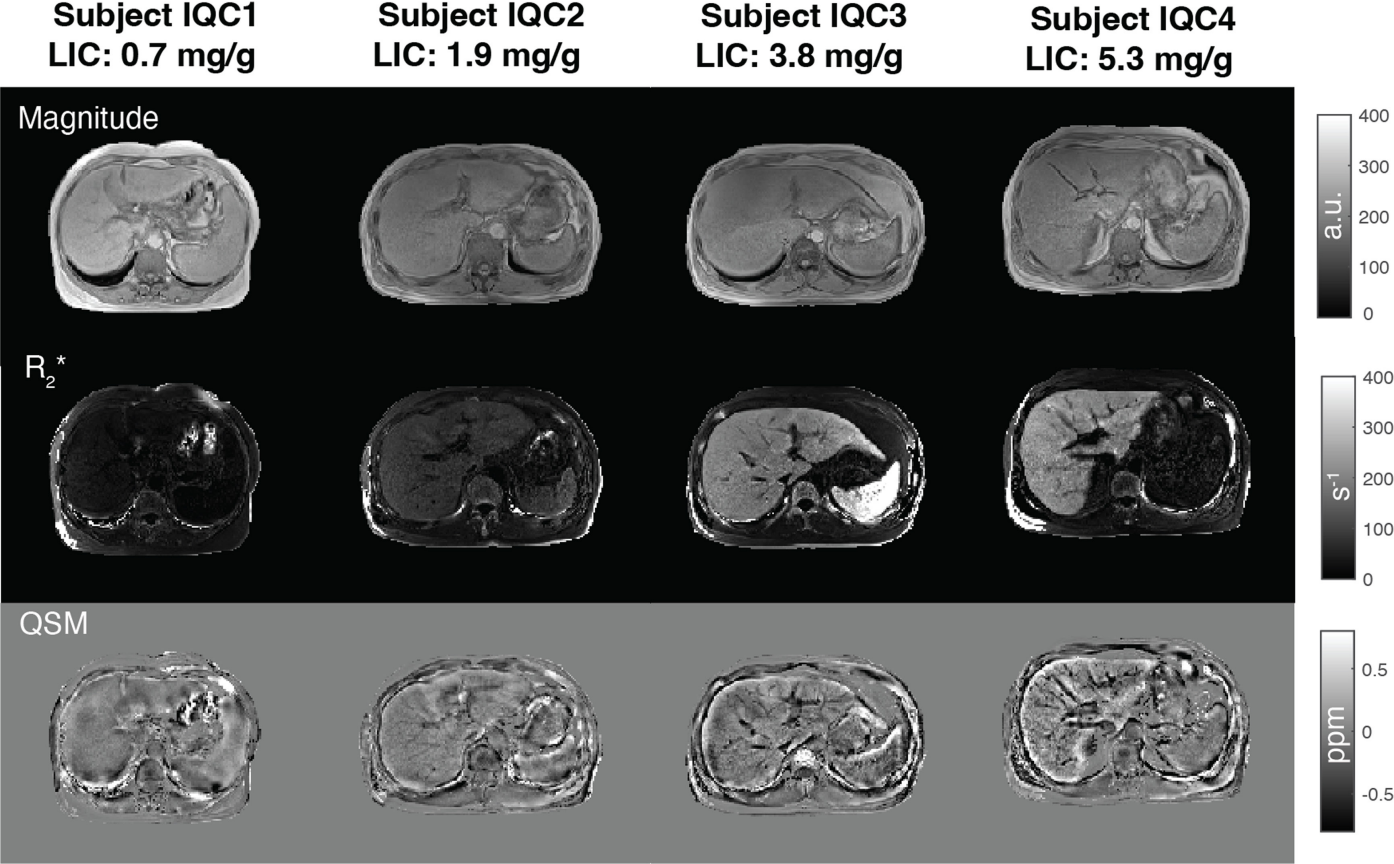
Magnitude, R_2_*, and QSM maps for four representative subjects from Iron Quantification Cohort (IQC). The R_2_* maps are generated as an output of the fat‐water separation algorithm. LIC measured by R_2_‐based FerriScan for these subjects are 0.7, 1.9, 3.8, and 5.3 mg/g dry tissue, respectively.

Figure 4 presents the PCS, DCS along with thresholded QSM maps (QSM < 0 and QSM > 0) for the same four subjects as in Figure 4. Notably, both PCS and DCS maps detect magnetic content that is not captured by the conventional QSM. Some vessel structures appear diamagnetic, while surrounding tissues are paramagnetic, a pattern consistently observed in both the QSM and source‐separated maps.

**FIGURE 4:**
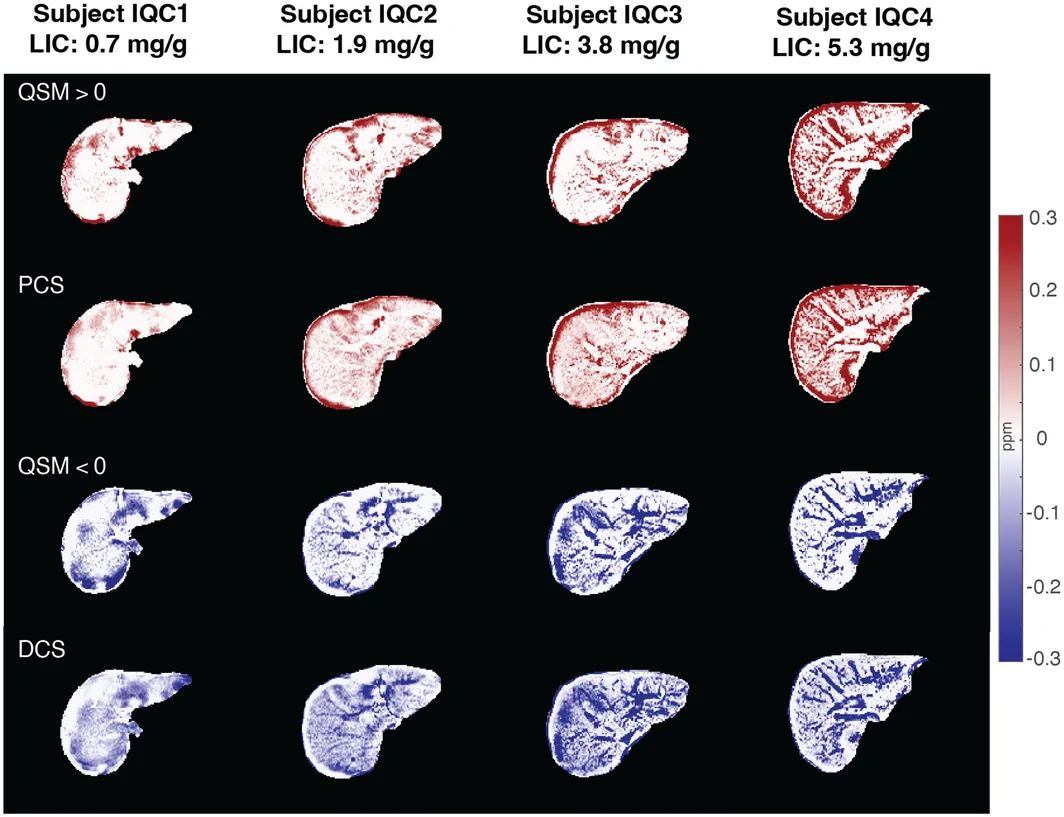
PCS, DCS, and thresholded QSM maps for the same four subjects shown in Figure 3. Source separation reveals paramagnetic and diamagnetic components, suggesting coexistence of iron and collagen.

Figure 5 shows the correlation between mean liver QSM, PCS, DCS, and LIC measured by FerriScan in 12 subjects. Mean liver QSM showed a weak correlation with LIC (*R* ^2^ = 0.28, *p* = 0.08), with a slope of 23.7 (mg/g)/ppm and an intercept of 1.12 ppm. After susceptibility source separation using DECOMPOSE‐QSM, the mean PCS exhibited a strong positive correlation with LIC (*R* ^2^ = 0.86, *p* < 0.05), with a slope of 48.6 (mg/g)/ppm and an intercept of −0.643. Meanwhile, the mean DCS demonstrated a weak negative correlation with LIC (*R* ^2^ = 0.21, *p* = 0.14), with a slope of −30.6 (mg/g)/ppm and an intercept of −0.432. These results suggest that PCS improves the iron sensitivity compared to conventional QSM.

**FIGURE 5:**
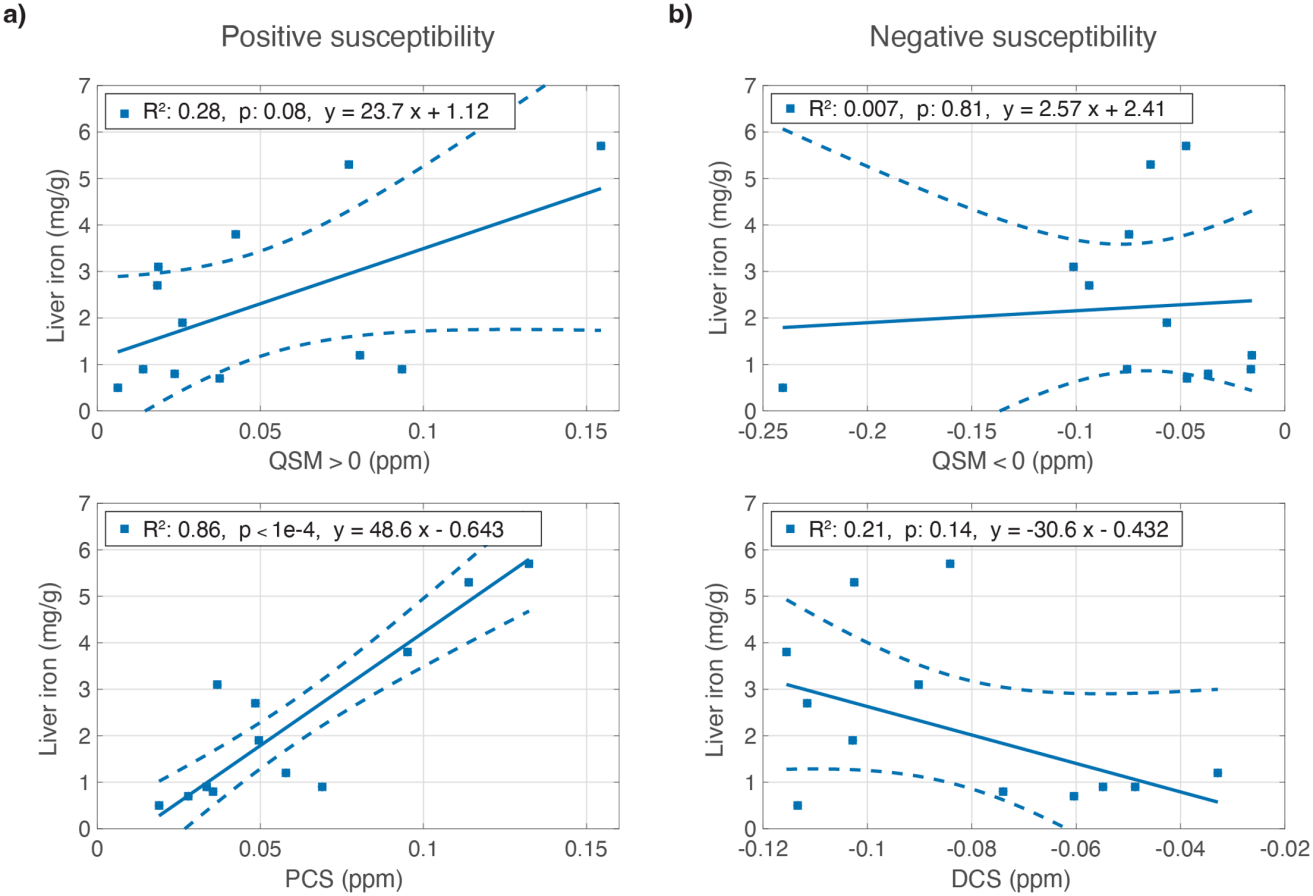
Scatter plots showing the relationship between FerriScan‐measured LIC and (a) QSM > 0 and PCS, and (b) QSM < 0 and DCS in 12 subjects. A strong positive correlation is observed for PCS (*R* ^2^ = 0.86, *p* < 0.05), suggesting it is a robust predictor of liver iron load. Dashed lines represent the 95% confidence intervals for the linear regression line.

### 3.3 Liver Fibrosis Assessment With DECOMPOSE‐QSM

Figure 6 illustrates the comparative analysis of QSM < 0 and DCS values for patients with fibrosis (FC dataset). All subjects in the FC dataset were diagnosed with fibrosis, with subject FC3 exhibiting the most severe disease, associated with Budd–Chiari syndrome. As increased diamagnetic susceptibility is expected in fibrosis, both QSM < 0 (FC1: −0.044 ppm; FC2: −0.038 ppm; FC3: −0.058 ppm) and DCS values (FC1: −0.050 ppm; FC2: −0.033 ppm; FC3: −0.057 ppm) were more negative in FC3. This finding suggests that DCS captures fibrosis‐related increases in diamagnetic content, consistent with conventional QSM.

**FIGURE 6:**
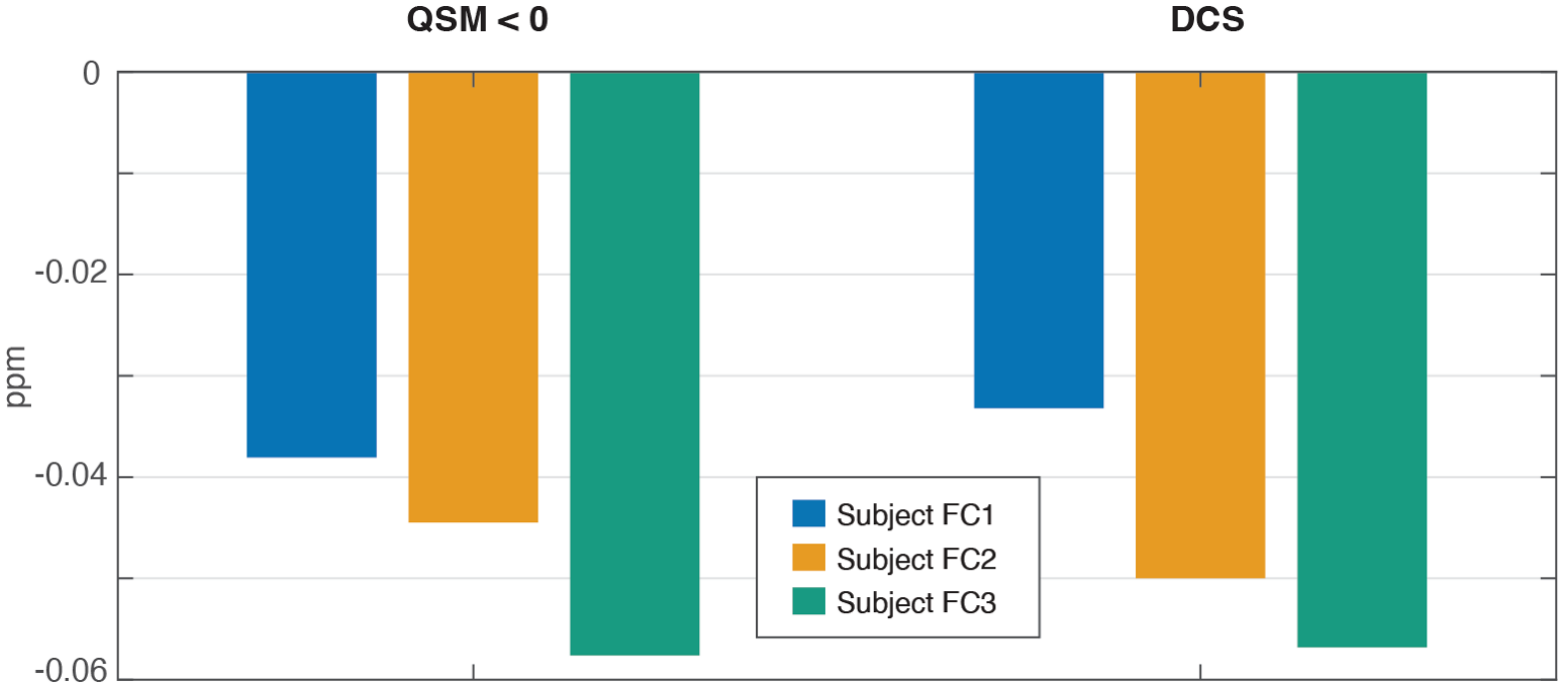
Comparison of QSM < 0 and DCS values for subjects in the fibrotic cohort. Subject FC3 exhibits the most advanced stage of fibrosis among the displayed subjects.

## 4 Discussion

Separation of paramagnetic and diamagnetic sources in the liver has the potential to differentiate iron overload from fibrosis. This capability holds significant clinical potential as a non‐invasive alternative to liver biopsy for both iron quantification and fibrosis detection. In this study, we employed the DECOMPOSE‐QSM method to resolve these sources across a spectrum of iron concentrations and fibrosis stages. The resulting PCS and DCS maps demonstrated superior contrast relative to conventional bulk susceptibility maps obtained through standard QSM reconstruction, possibly addressing the bulk averaging that often masks coexisting pathologies.

A fundamental challenge in abdominal QSM is the confounding effect of fat chemical shift. While we utilized a graph‐cut‐based fat‐water separation algorithm to generate water‐only and fat‐only images, this algorithm operates under the assumption that water‐only image phase is a linear function of echo time. This assumption is incompatible with the nonlinear phase behavior inherent to the biophysical model of DECOMPOSE‐QSM. To preserve this critical nonlinear information, we proposed a residual fat‐subtraction strategy to obtain nonlinear‐phase water images, wherein the fat‐only complex image is subtracted from the original multi‐echo complex data. This approach maintains the integrity of the underlying phase perturbations while successfully isolating the water signal of interest.

Using fat‐saturated acquisitions as a reference, our qualitative and quantitative comparisons demonstrated that using nonlinear‐phase water images is better fat‐removal strategy for DECOMPOSE‐QSM compared to using linear‐phase water images. Qualitatively, nonlinear‐phase water images show highly similar paramagnetic and diamagnetic distributions to the fat‐saturated reference, whereas linear‐phase water images display diminished paramagnetic signal intensity and increased diamagnetic signal. Quantitatively, Bland–Altman analyses confirmed that nonlinear‐phase water images maintain close agreement with the reference, yielding minimal mean biases for PCS and DCS across both datasets. Although fat‐saturated acquisitions were used as the reference in this study, caution is warranted, as imperfect fat suppression may introduce phase errors that could compromise the accuracy of the reference and, in turn, the quantitative comparisons.

A key consideration in our approach is the reliance on accurate fat‐water separation for both linear‐ and nonlinear‐phase water images. Errors in the fat separation step can introduce phase artifacts, leading to biases in QSM and subsequent PCS or DCS measurements. In our study, 7 of the 19 subjects in the IQC dataset were excluded due to fat‐water swaps in the liver, reducing the final sample size to 12 subjects. This observation highlights the potential value of developing integrated fat‐separation and QSM algorithms, which could enhance robustness in clinical liver imaging.

Moreover, breath‐held acquisitions naturally constrain scan time and spatial resolution, which can influence susceptibility measurements [59], while SNR limitations may affect fat–water separation precision. Future implementations using motion‐compensated or free‐breathing sequences offer a promising pathway toward higher SNR and more precise source separation, further expanding the applicability of this method in clinical practice.

Another key challenge in liver QSM, and consequently in source separation, is defining a reliable zero‐susceptibility reference. This requires identifying a region whose magnetic susceptibility remains stable across subjects, enabling meaningful inter‐subject comparisons. In our study, fat was used as the zero‐susceptibility reference; however, it is also possible to employ alternative references, including the aorta [30], subcutaneous fat alone [25, 26], or external reference objects [31]. Since the chosen reference affects the baseline of the entire susceptibility map, it can systematically bias all measurements. We observed that vessel‐like structures in the liver became more diamagnetic with increasing iron concentration, consistent with earlier reports [30]. This pattern may arise from reference‐related bias: if the reference region contains errors or undergoes susceptibility changes itself, the calculated vessel susceptibilities may be shifted toward more negative values. Furthermore, although fat has a higher magnetic susceptibility than water‐based tissue and may therefore contribute positively to the measured susceptibility [33, 60, 61, 62, 63], this potential contribution was not modeled separately in the present study and may have introduced a bias, particularly in participants with higher hepatic fat fractions.

Our results on LIC correlation suggest that incorporating susceptibility source separation enhances sensitivity to liver iron quantification. Although previous studies have reported strong correlations between QSM and LIC [25, 26, 31], in our work, conventional QSM showed a weak correlation with LIC. After applying DECOMPOSE‐QSM, the mean PCS values showed a strong positive correlation with LIC (R^2^ = 0.86, *p* < 0.05), suggesting susceptibility source separation via DECOMPOSE‐QSM enhances specificity to iron while potentially enabling independent characterization of fibrotic tissue. In this study, we used $R_{2}$‐based FerriScan results as the reference for LIC measurements. Previous extensive calibration and validation studies have shown that liver fibrosis does not introduce a significant systematic bias into $R_{2}$‐based LIC measurements [18, 20]. Future work should include a direct LIC reference, such as liver biopsy or SQUID‐based measurements, to further validate and strengthen our findings.

While DECOMPOSE‐QSM was used for susceptibility source separation in this study, other source separation methods have also been proposed for GRE‐only data [41, 42, 43, 44, 45] These methods assume a linear susceptibility‐relaxation relationship and therefore require a relaxometric coefficient, with reported calibrations derived mainly from brain tissue. A recent ex vivo liver study optimized a liver‐specific relaxometric coefficient to maximize discrimination of histologic fibrosis stages [46]. To explore this approach in our in vivo liver data, we additionally evaluated QSM‐ARCS [44] using the reported liver specific coefficient as the initial value for adaptive relaxometric coefficient estimation (see Supplementary Materials). However, because this coefficient was derived from ex vivo liver at 3 T and has not been validated for in vivo liver imaging at 1.5 T, these results should be interpreted with caution.

Our comparative analysis within the fibrotic cohort suggests that source‐separated diamagnetic values exhibit trends consistent with fibrosis severity, similar to those observed with QSM < 0. These findings indicate that DCS captures fibrosis‐related diamagnetic changes while maintaining consistency with conventional QSM measurements. However, several limitations warrant consideration, including the small fibrosis sample size (*n* = 3) and reliance on clinical rather than histopathological diagnosis. Future studies incorporating spatially matched biopsy references and larger cohorts spanning all fibrosis stages are needed to further validate DCS as a potential clinical biomarker.

## 5 Conclusion

Our findings suggest that DECOMPOSE‐QSM potentially enables separation of paramagnetic and diamagnetic susceptibility sources within a voxel in the liver. By disentangling these opposing components, source‐separated paramagnetic susceptibility showed an improved correlation with liver iron concentration compared with conventional QSM for the cases of normal to moderate iron overload. These results highlight the potential of susceptibility source separation to improve sensitivity and specificity for iron quantification. In addition, preliminary observations indicate that source‐separated diamagnetic susceptibility captures fibrosis‐related susceptibility changes and demonstrates trends consistent with conventional QSM, supporting its potential role in the noninvasive characterization of liver fibrosis.

## Author Contributions

**Asli Alpman:** methodology, software, formal analysis, investigation, writing — original draft, visualization. **Jingjia Chen:** methodology, software. **Jorge Elías Junior:** fibrotic cohort data. **Carlos Ernesto Garrido Salmon:** fibrotic cohort data. **Fuhua Yan:** healthy control and iron quantification cohort data. **Hongjiang Wei:** healthy control and iron quantification cohort data. **Chunlei Liu:** conceptualization, methodology, resources, writing — review and editing, supervision, project administration, funding acquisition.

## Funding

This work was supported by Weill NeuroHub.

## Conflicts of Interest

The authors declare no conflicts of interest.

## Supporting information


**Figure S1:** QSM‐ARCS results are shown for four representative subjects from the Iron Quantification Cohort (IQC), spanning liver iron concentrations (LIC) of 0.7, 1.9, 3.8, and 5.3 mg/g dry tissue, respectively, as measured by R_2_‐based FerriScan. The corresponding susceptibility maps ($\chi^{+},\chi^{-}$, and χ maps) derived using QSM‐ARCS are shown for each subject. There are some apparent “striping” artifacts in the maps, indicated by the green arrows.
**Figure S2:** Scatter plots show FerriScan‐measured LIC versus (a) χ>0 and χ+, and (b) χ<0 and χ− in 12 subjects. Solid lines indicate linear regression fits and dashed lines the corresponding 95% confidence intervals. For positive susceptibility, QSM‐ARCS source separation reduced the number of outliers. For negative susceptibility, source separation resulted in a strong association with LIC. Because of fat referencing and streaking‐artifact removal, the separated susceptibility components are not strictly sign‐constrained.

## Data Availability

The data that support the findings of this study are available from the corresponding author upon reasonable request.
