# Supplementary material for: Separation of Diamagnetic and Paramagnetic Susceptibility Sources in Liver Using DECOMPOSE‐QSM
Source: NMR Biomed. 2026 Nov 6;39(12):e70434. doi: 10.1002/nbm.70434 (PMC13743794; doi:10.1002/nbm.70434)
Supplement: Supplementary file 1 [file NBM-39-e70434-s001.docx]

## Supplementary for Separation of Diamagnetic and Paramagnetic Susceptibility Sources in Liver Using DECOMPOSE-QSM

**Supplementary Figure 1.** QSM-ARCS results are shown for four representative subjects from the Iron Quantification Cohort (IQC), spanning liver iron concentrations (LIC) of 0.7, 1.9, 3.8, and 5.3 mg/g dry tissue, respectively, as measured by R_2_-based FerriScan. The corresponding susceptibility maps ($\chi^{+}, \chi^{-}$and $\chi$maps) derived using QSM-ARCS are shown for each subject. There are some apparent “striping” artifacts in the maps, indicated by the green arrows.

**Supplementary Figure 2.** Scatter plots show FerriScan-measured LIC versus (a) $\chi>0$ and $\chi^{+}$, and (b) $\chi<0$ and $\chi^{-}$in 12 subjects. Solid lines indicate linear regression fits and dashed lines the corresponding 95% confidence intervals. For positive susceptibility, QSM-ARCS source separation reduced the number of outliers. For negative susceptibility, source separation resulted in a strong association with LIC. Because of fat referencing and streaking-artifact removal, the separated susceptibility components are not strictly sign-constrained.
